# Runx2 and Nell-1 in dental follicle progenitor cells regulate bone remodeling and tooth eruption

**DOI:** 10.1186/s13287-022-03140-3

**Published:** 2022-09-30

**Authors:** Li Zeng, Hong He, Mingjie Sun, Xinyi Gong, Mengqi Zhou, Yaya Hong, Yongjia Wu, Xuepeng Chen, Qianming Chen

**Affiliations:** 1 Stomatology Hospital, School of Stomatology, Zhejiang University School of Medicine, Hangzhou, 310006 Zhejiang China; 2 Clinical Research Center for Oral Diseases of Zhejiang Province, Key Laboratory of Oral Biomedical Research of Zhejiang Province, Cancer Center of Zhejiang University, Hangzhou, 310006 China

**Keywords:** Tooth eruption, RUNX2/Runx2, NELL-1/Nell-1, Dental follicles (DFs), Dental follicle progenitor cells (DFPCs), Neural crest, Osteoblast, Osteoclast, Bone remodeling, Noncoding RNAs (ncRNAs), Competitive endogenous RNAs (ceRNAs)

## Abstract

Dental follicles are necessary for tooth eruption, surround the enamel organ and dental papilla, and regulate both the formation and resorption of alveolar bone. Dental follicle progenitor cells (DFPCs), which are stem cells found in dental follicles, differentiate into different kinds of cells that are necessary for tooth formation and eruption. Runt‐related transcription factor 2 (Runx2) is a transcription factor that is essential for osteoblasts and osteoclasts differentiation, as well as bone remodeling. Mutation of Runx2 causing cleidocranial dysplasia negatively affects osteogenesis and the osteoclastic ability of dental follicles, resulting in tooth eruption difficulties. Among a variety of cells and molecules, Nel-like molecule type 1 (Nell-1) plays an important role in neural crest-derived tissues and is strongly expressed in dental follicles. Nell-1 was originally identified in pathologically fused and fusing sutures of patients with unilateral coronal synostosis, and it plays indispensable roles in bone remodeling, including roles in osteoblast differentiation, bone formation and regeneration, craniofacial skeleton development, and the differentiation of many kinds of stem cells. Runx2 was proven to directly target the Nell-1 gene and regulate its expression. These studies suggested that Runx2/Nell-1 axis may play an important role in the process of tooth eruption by affecting DFPCs. Studies on short and long regulatory noncoding RNAs have revealed the complexity of RNA-mediated regulation of gene expression at the posttranscriptional level. This ceRNA network participates in the regulation of Runx2 and Nell-1 gene expression in a complex way. However, non-study indicated the potential connection between Runx2 and Nell-1, and further researches are still needed.

## Introduction

Cranial neural crest cells (CNCCs), which are important in craniofacial tissue development [1], can differentiate into dental follicles. Dental follicles (DFs), which surround the enamel organ and dental papilla, play an important role in tooth eruption by regulating bone remodeling near erupting teeth. Dental follicle progenitor cells (DFPCs), a group of undifferentiated ectomesenchymal cells found in DFs, express stem cell markers and can differentiate into many kinds of cells that are necessary for tooth eruption. To control tooth eruption in a strict spatiotemporal manner [2], alveolar bone remodeling is meticulously controlled by dental follicles.

Tooth eruption refers to the process that includes tooth germ calcification within the jaw, breakthrough in the oral epithelia and exposure in the oral cavity; the tooth gradually reaches its functional position and achieves occlusal contact with the opposite tooth. Since teeth are surrounded by bone, eruption depends on the precise regulation of bone remodeling. The mechanism underlying tooth eruption mainly involves two processes [3], namely formation and resorption of alveolar bone, and these processes are regulated by dental follicles. Dental follicles themselves exhibit polarity: the crown area of dental follicles can regulate the formation of osteoclasts and the absorption of alveolar bone that are necessary for tooth eruption, while the root side can regulate the formation of alveolar bone that is crucial for eruption [4]. Various regulatory mechanisms that occur near an erupting tooth affect its occlusal and apical sides.

Runx2 is a transcription factor that is essential for the differentiation of osteoblasts and osteoclasts and the bone remodeling. In addition, haploinsufficiency of Runx2 had been proven to have a causal link with cleidocranial dysplasia (CCD). Runx2 promotes the differentiation of osteoclasts via the RANK/RANKL [5] and RANKL/OPG [6] pathways. Genetic abnormality of Runx2 interferes with the functions of periodontal ligament cells (PDLCs) and dental follicle cells, which lose their ability to regulate the differentiation of osteoblasts and osteoclasts and fail to control bone remodeling, especially on the apical side of an erupting tooth.

Nel-like molecule type 1 (Nell-1), whose expression was originally shown to be increased in pathologically fused and fusing sutures of patients with unilateral coronal synostosis (UCS), plays indispensable roles in bone remodeling, including roles in osteoblast differentiation, bone formation and bone regeneration, and Nell-1 is also important for craniofacial skeleton development. Nell-1 has specificity for the craniofacial region and was strongly expressed in dental follicles [7]. Moreover, Nell-1 can promote the differentiation of different kinds of stem cells. Runx2 targets the Nell-1 gene and directly regulates its expression by binding to osteoblast-specific binding element 2 (OSE2) sites in the promoter of Nell-1.

Studies on short and long regulatory noncoding RNAs have revealed the complexity of RNA-mediated regulation of gene expression during transcription of DNA and translation of proteins [8]. MicroRNAs (miRNAs) function in the posttranscriptional regulation of gene expression, suppress the expression of target mRNAs [9, 10] and participate in several biological functions, such as embryogenesis, organogenesis, cell differentiation, developmental timing and apoptosis [11]. Long noncoding RNAs (lncRNAs) [12] and circular RNAs (circRNAs) [13] act as miRNA sponges to regulate the expression of genes by competitively binding to miRNA response elements (MREs) to exert important biological functions. This complex ceRNA network could affect the expression of Runx2 and Nell-1 in posttranscriptional level.

This review summarizes the process of tooth development and eruption, and the function and regulatory pathway of Runx2 and Nell-1 generally. We also review some current studies on ceRNA network associated with Runx2 and Nell-1, and put forward the argument that Runx2/Nell-1 axis may act as one of the important regulatory pathway during tooth eruption in DFPCs based upon existing evidence. At the same time, we summarize the deficiency of existing researches and propose the direction of future researches.

### Tooth eruption

The precursor cells of the cranial neural crest (CNCC), which are generated in the outer edge of the neural fold between the neural epiblast and its surface, move toward the ventrolateral region and are distributed in the branchial arch as osteogenic neural crest cells and cranial nerve ganglion [14]. CNCCs are important in craniofacial tissue development [1]. Notably, CNCCs can be derived from multiple craniofacial tissues, including the skeleton, cartilage, loose connective tissues, ganglia, and nerves [14]. Ectoblastic mesenchyme originating from the neural crest differentiates into dental follicles (DFs) and dental papilla, and these tissues cooperate with enamel organs, which originate from the oral epithelium, to participate in tooth development and eruption.

Dental follicles (DFs) are loose connective tissue and surround the dental papilla and enamel organ and contain stem cells and a precursor cell subpopulation that can develop into periodontal tissue [4]. DFs contain undifferentiated ectomesenchymal cells known as dental follicle progenitor cells (DFPCs) [15], which express the stem cell markers nestin, Notch1, and STRO-1 [16]. These mesenchymal progenitor cell populations were shown to be positive for parathyroid hormone-related peptide (PTHrP), glioma-associated oncogene homolog 1 (Gli1) and Osterix (Osx) expression [17–19] in CreER mouse models.

**Fig. 1:**
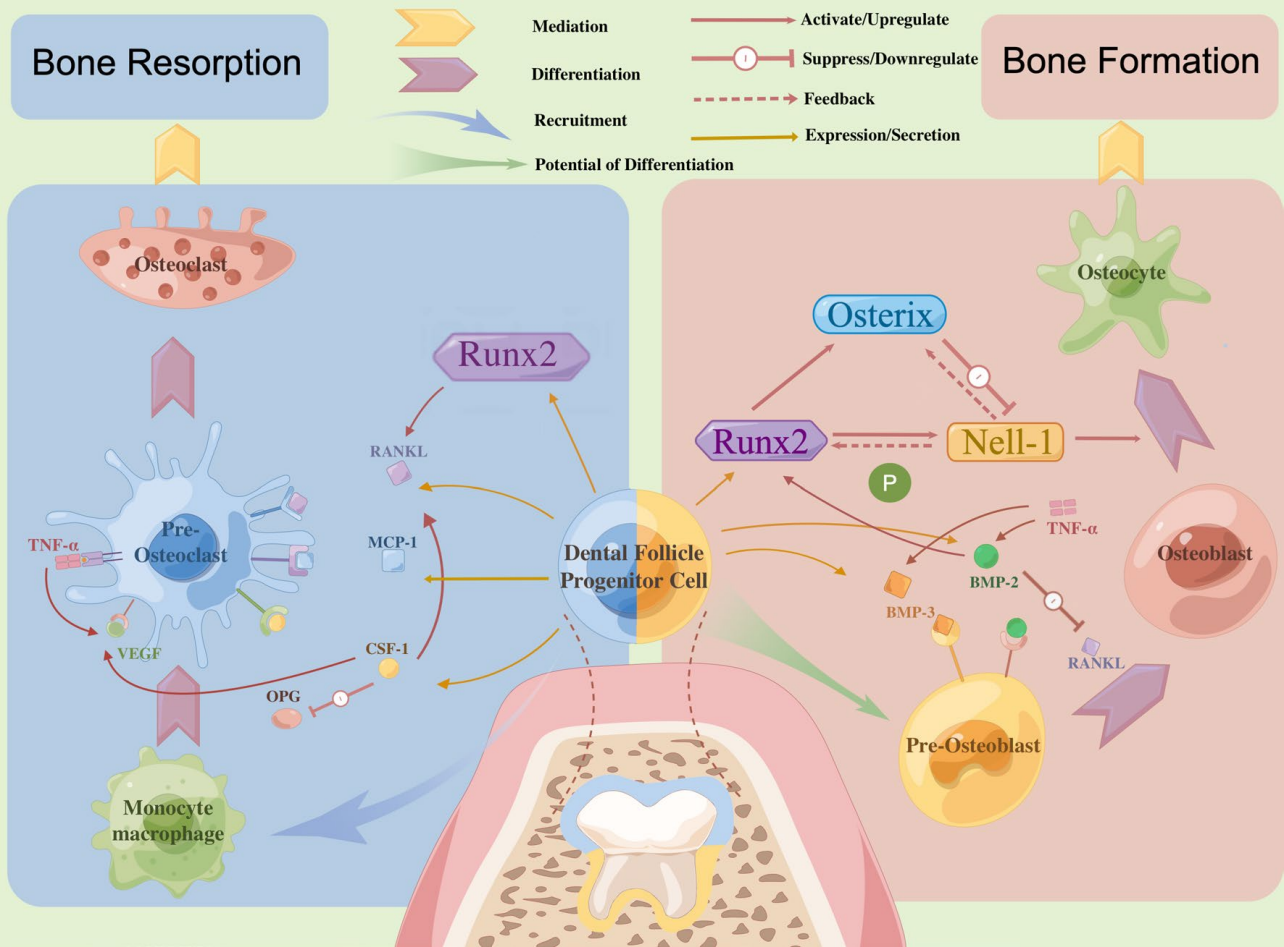
Encircling dental papilla and enamel organ, dental follicles with two polarity plays an indispensable part in tooth eruption by regulating bone remodeling around erupting tooth in a temporospatial manner. Dental follicle Progenitor cells (DFPCs) in the crown side regulate the absorption of alveolar bone by stimulating the proliferation and differentiation of osteoclasts through many cytokines and pathways, among which RANKL and CSF-1 being known to accelerate the formation of osteoclast are critical, while in the root side, DFPCs regulate the deposition of alveolar bone by stimulating the proliferation and differentiation of osteoblasts through coordinating with many molecules. In the process of deposition of trabecular bone, the Runx2/Nell-1 axis may act as one of the key regulatory pathways in DFPCs, and Osterix contribute to a subtle balance of regulatory effects on Nell-1 transcription with Runx2

Because tooth germ develops and is buried in the alveolar bone, the process of tooth eruption relies on the spatiotemporal regulation of bone remodeling [20]. The mechanism underlying tooth eruption mainly involves two processes [3]: the formation of a tooth eruption canal, which includes the root absorption of deciduous teeth and absorption of alveolar bone, and active tooth eruption, which includes the development of a permanent tooth embryo and interactions among the periodontal membrane, periapical tissue and pulp [4]. Dental follicles accumulate cells with osteoclastic ability in the coronal area to absorb overlying alveolar bone and the roots of the deciduous tooth when the root starts to form; at the same time, the permanent tooth begins to move in the occlusal direction and bone deposition occurs on the root side (see Fig. 1).

Many studies have demonstrated that the regulation of dental follicles, multiple molecules, osteoblasts, osteoclasts, and alveolar bone are necessary for tooth eruption. Both alveolar bone formation and resorption, which are necessary for tooth eruption, are controlled by dental follicles near an erupting tooth [4], especially DFPCs (see Fig. 1). As reported in the 1980s [21], tooth germ itself such as tooth pulp or tooth root is not a necessary factor in the regulation of the tooth eruption process, and thus, researchers have examined dental follicles and enamel organs when studying the regulatory mechanisms of tooth eruption. Alveolar bone remodeling occurs in the context of a meticulous arrangement of DFs and is strictly regulated in a spatiotemporal manner [2] during tooth eruption.

Dental follicles participate in the resorption of alveolar bone by recruiting mononuclear cells, especially osteoclast precursors, to 1/3 of the area of the crown side to promote osteoclast maturation to facilitate tooth eruption. Receptor activator for nuclear factor-κ B ligand (RANKL) [22] and colony-stimulating factor 1 (CSF-1) [23], which are expressed by cells of the osteoblast lineage, are two important molecules that are closely related to the differentiation and maturation of osteoclasts. Upregulation of receptor activator for nuclear factor-κ B (RANK) protein expression can also be mediated by CSF-1 in preosteoclast cells to further reinforce the expression of RANK/RANKL signaling molecules and enhance the survival and proliferation of preosteoclast cells [2]. Matrix metalloproteases (MMP) and macrophages also participate in this process. In addition, the reduced enamel epithelium of enamel organs can interact with DFCs to aggregate osteoclasts and promote alveolar bone remodeling [24].

The root side of the dental follicles also participates in the regulation of alveolar bone formation. DFPCs have the potential of osteogenic differentiation and can produce mineralized matrix. The formation of alveolar bone on the root side serves as the major motive force to promote the movement of tooth along the eruption channel under the alveolar bone with less resistance to eruption [25, 26]. Though with the normal formation of eruption pathway, delayed tooth eruption was observed for a lack of eruption force if bone formation was prevented in the local region, suggesting that alveolar bone formation is a necessary factor for tooth eruption [27]. The transcription factors Runx2 and BMP-2 are the most essential factors on the apical side of an erupting tooth, and these factors function mainly in the deposition of trabecular bone in the root formation zone [28]. Other molecular mechanisms underlying the osteogenic differentiation of DFPCs include Wnt, parathyroid hormone 1 receptor (PTH1R), Notch, and BMP [29]. Coupled with Runx2 [30], odontogenic ameloblast-associated protein (ODAM) promotes the osteogenic differentiation of DFPCs, thus contributing to alveolar bone formation during tooth eruption. Under certain conditions, these precursor cells can differentiate into osteoblasts, periodontal membrane cells or cementoblast-like cells and form periodontal membrane, cementum and inherent alveolar bone in later stages of tooth development [31].

The dental epithelia and the neural crest-derived ectoblastic mesenchyme interact with each other [32] via mechanisms that are precisely genetically controlled to develop the tooth organ. The direction and speed of endosseous eruptions are determined by genetic and local environmental elements instead of the osteoclastic canal, and the local environmental elements play the leading role. Tooth eruption actually has a reciprocal relationship with tooth formation, which allows permanent teeth to erupt when the root reaches 2/3 to 3/4 of its ultimate length. Numerous biological molecules, including extracellular matrix molecules, transcription factors, and growth factors, participate in regulating tooth formation and cell differentiation [33]. A series of physiological processes of tooth formation and eruption, which are disrupted in patients with many hereditary syndromes and genetic mutations [34], are precisely and strictly controlled by genetic and local environmental factors [35]. When the processes of tooth eruption and formation are both influenced by genetic mutations, the appearance of complex clinical problems becomes a difficult issue for many clinicians and requires multidisciplinary operations.

### Runx2

Runx2 is a transcriptional factor that is critical for the development of osteoblastic cells and osteogenesis. CCD, which is characterized by abnormal bone development, has been considered to occur as a result of Runx2 haploinsufficiency [38]. Through exon sequencing analysis of healthy people and patients with CCD with unique clinical manifestations, Ge et al. [36] found a new mutation in the Runx2 (c.634T > G, p. T212P) gene. Dental performance of patients with CCD includes abnormalities in tooth genesis, including supernumerary tooth formation, abnormal morphology of permanent teeth, especially that of the roots, and tooth eruption, including suspended or unsuccessful eruption of permanent teeth and early uncovering of primary teeth. Runx2-heterozygous mice displayed skeletal development symptoms similar to those of human patients with CCD, including cranial and clavicular hypoplasia, unclosed fontanelles in the anterior and posterior skull, wormian bones in multiple sutures, and hypoplastic parietal and interparietal skull skeleton [37].

Runx2 is one of the most critical transcriptional molecules in DFs, and studies indicated that Runx2 mutation damaged the normal osteogenic and osteoclastic functions of DFs and thus resulted in the delayed or arrested tooth eruption in CCD. DFCs in patients with CCD showed significantly reduced abilities to induce osteoclast and osteoblast differentiation, as well as reduced matrix degradation, downregulated Runx2 expression, lower SATB2 expression, and higher expression of miR-31, whose transcription is inhibited by Runx2, when compared with healthy DFCs. Arrested tooth eruption and osteoclasts with low activity in DFs were observed in newborn mice administered siRunx2 in vivo [36]. Yoda et al. [38] found impaired recruitment of osteoclasts in the eruption pathway in delayed teeth eruption of mouse model and suggested that this is one of the cellular mechanisms of delayed tooth eruption in patients with CCD. Lossdörfer et al. [39] found human periodontal ligament cells (hPDLCs) from CCD showed a reduced capacity to induce the differentiation of active osteoclasts. Many studies of DFCs from patients with CCD have reported disturbed osteoclast-inductive signaling in DFCs, which these authors suggest could be responsible for delayed tooth eruption in patients with CCD [36, 40, 41]. On the other hand, Runx2 mutation can reduce the osteogenic capacity of DFCs through inhibiting osteoblast-associated genes, including Runx2, alkaline phosphatase (ALP), osterix (Osx), osteocalcin (OCN) and Collagen Type I α 1 (Col Iα1), thereby disturbing alveolar bone formation, which serves as a motive force for tooth eruption [42]. Experiments involving DFCs and PDLCs from an 11-year-old patient with CCD [43] demonstrated that the regulatory function of PDLCs and DFCs is disrupted by Runx2 mutation, which in turn negatively affects the differentiation of osteoblasts and osteoclasts as well as skeletal remodeling. These effects might partially cause the pathological characteristics of CCD, including delayed or arrested permanent tooth eruption and retention of deciduous teeth, as suggested by the authors.

Multiple studies, including molecular biology studies, animal model studies, and clinical studies, have demonstrated that Runx2 is important in osteoblast differentiation, bone formation, osteoclast development in DFs and PDL, dental lamina degeneration, bone resorption, bone remodeling and chondrocyte maturation. As for osteoclast development and bone resorption, reductions in the RANKL/OPG and RANKL/RANK ratios in the DFCs of patients with CCD were observed, indicating inhibition of osteoclast generation signaling in DFC-CCD [40]. The RANKL/RANK/OPG signaling pathway is an important signaling pathway that regulates the differentiation, maturation and function of osteoclasts [44]. In addition to the RANK/RANKL [5] and RANKL/OPG [6] pathways, multiple proteins of the matrix metalloproteinase (MMP) family also participate in tooth eruption [45, 46]. Among MMP family members, MMP9 was confirmed to be a downstream target of Runx2 [47] and plays a leading role in bone resorption [48] as well as osteolysis [49]. Collectively, both animal model studies and cell or molecular biology studies suggest that Runx2 haploinsufficiency or mutation leads to impaired RANKL/OPG, MMP9, and RANK/RANKL signaling during osteoclastogenesis, which partly results in arrested tooth eruption in patients with CCD [36].

As for osteoblast differentiation and bone formation, Runx2 binds to the osteoblast-specific binding elements 2 (OSE2) sites in the promoter regions of many downstream target genes to regulate osteoblastic transcription, in which α1 type I collagen (Col1-α1), bone sialoprotein (Bsp), osteopontin (Op), osteocalcin (Ocn), osterix (Osx), and Nell-1 were well-studied [50]. However, animal knockout models of Col1-a1 [51], Bsp [52], Op [53], and Ocn [54] in mice have not yielded significant developmental defects similar to Runx2 deficiency. Nell-1 knockout mice model showed different result [55]. Genes including Osx lack the verification of animal knockout model. A heterozygous null mutation at the α1 type I collagen (Col1-a1) genes in Mov-13 mice model showed the long bones defects with reduced mechanical and material properties instead of cranial bones, also representing a model of human osteogenesis imperfecta type I (OI-I) [51]. Delayed tooth eruption or hypoplasia in cranial bone did not show in the animal model, because type I collagen is thought to make an important contribution to the structure and function of long bones instead of cranial bones [51]. BSP − / − mice displayed a reduced body and long bone growth, but have a high trabecular bone mass accompanied by low bone turnover, while defects in cranial bones or tooth eruption difficulties did not shown in the study [52]. Study [53] observed that trabecular bones in the midsagittal plane of the metaphyseas in the tibiae became sparse and reduction in quantification of the fractional trabecular bone volume (BV/TV) in OPN − / − mice, which is different with the phenotype in Runx2 knockout animals. Osteocalcin-deficient mice [54] developed a phenotype marked by higher bone mass and bones of improved functional quality, including increased cortical thickness and density, an increase in the width of the diaphysis and more cancellous bone in long bones. However, phenotype similar with CCD did not observed in cranial and alveolar bones.

### Nell-1

**Fig. 2:**
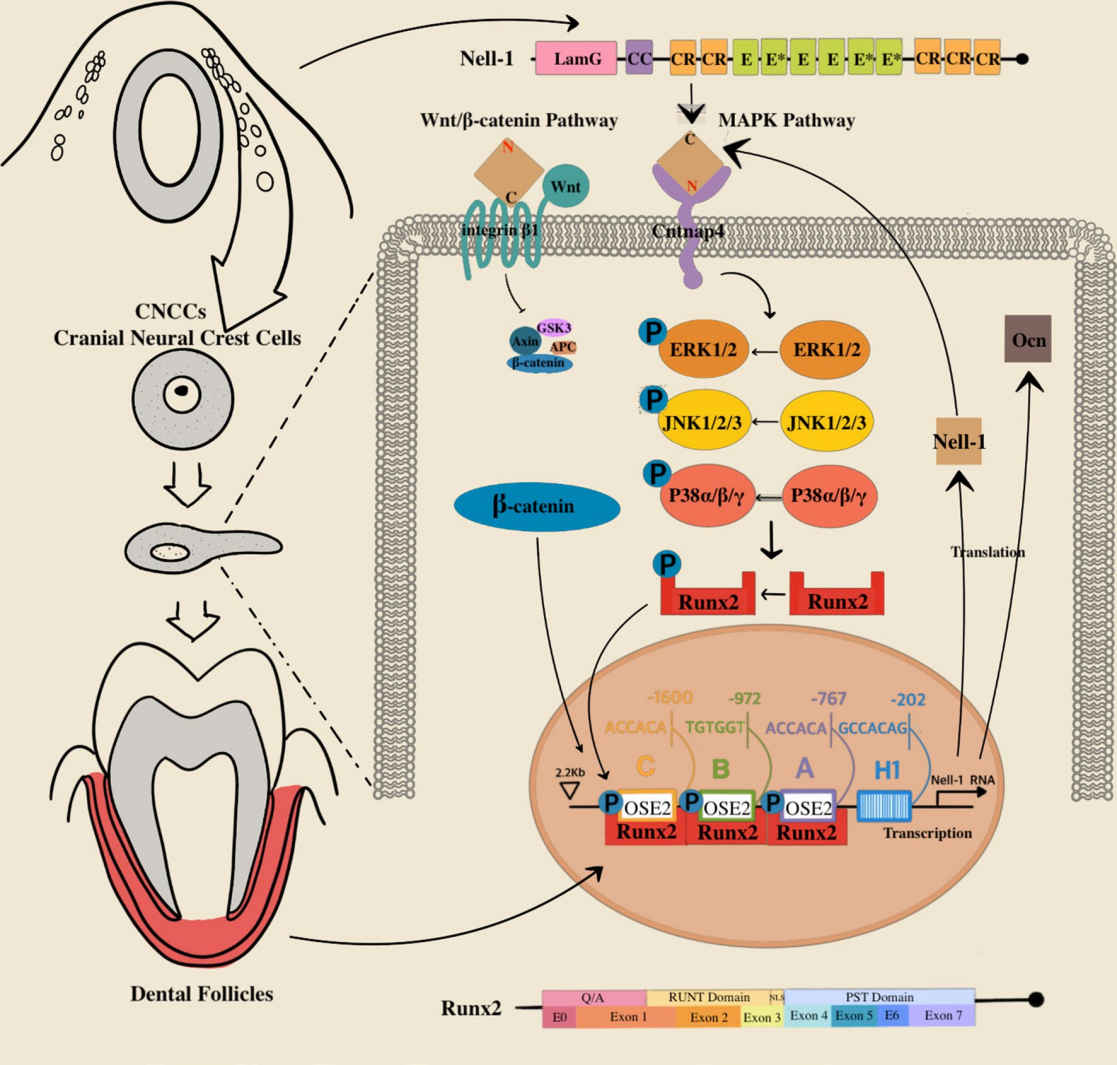
Nell-1’s expression take precedence in CNCCs (cranial neural crest cells), being of great importance in craniofacial tissue development. Nell-1 consists of extremely conservative modules including 5 CR (chordin‐like cysteine‐rich) motifs, a NH2‐terminal TSP‐1 (thrombospondin‐1)‐like motif, a coiled-coil (CC) module, a secretory signal peptide, a laminin G (LamG) module, and 6 EGF (epidermal growth factor)‐like domains (*E*) including Ca2+-binding EGF-like domains (E*). Runx2 consists of glutamine/alanine-rich region (*Q*/*A*); runt homology domain (RUNT); nuclear-localization signal (NLS); proline/serine/threonine-rich region (PST) which is 7 exon. Runx2 target and regulate Nell-1 directly through binding to OSE2 (osteoblast-specific binding element 2) sites in promoter of Nell-1. The Wnt/*β*-catenin and MAPK signaling pathways have leading role in osteogenesis mediated by Nell-1. Nell-1 stimulates JNK, ERK and p38 in MAPK signaling pathway preferentially and thereby boosts the phosphorylation of Runx2, which positively and circularly activates the expression of Nell-1 and Ocn by matching to the OSE2 elements in promoters. Besides, the active *β*-catenin in Wnt/*β*-catenin signaling is also upregulated and nuclear translocated by Nell-1

Functioning in combination with nuclear proteins, Nell-1 was originally found to be overexpressed in pathologically fused and fusing sutures of patients with UCS [56, 57]. Nell‐1 consists of 5 CR motifs, an NH2‐terminal TSP‐1‐like motif, a secretory signal peptide, and 6 EGF‐like domains, which can be phosphorylated by protein kinase C [58] (see Fig. 2). Nell-1 is associated with nonsyndromic UCS in humans, and it was overexpressed in pathologically premature fused and fusing coronal sutures [56]. A transgenic model of human craniosynostosis (CS) showed that the upregulation of Nell‐1 expression is the cause of UCS and that the development of craniofacial cartilage and bone cannot continue without Nell-1 [59].

Furthermore, Nell-1 is preferentially expressed in neural crest-derived tissues, including mesenchymal cells and differentiated osteoblasts of pathological fusing or fused coronal sutures in patients with UCS, suggesting its specificity for the craniofacial region [56]. Its preference for the craniofacial area suggests that it is a pivotal regulator that is necessary for CNCCs to achieve their complete osteoplastic potential and for normal craniomaxillofacial ossature growth [33]. Reduced bone mineral density (BMD) and decreased bone volume (BV) were observed in the CNCC original craniofacial bones of embryonic and newborn Nell-1^Wnt1^KO mice with full penetrance when Nell-1 was knocked out in cells expressing Wnt1. Except for a number of severe cases, analogous dysgenesis of craniofacial bone was also observed in most of the Nell-1^CMV^KO mice. These findings further indicated a leading role of Nell-1 in craniomaxillofacial bone tissues. The osteoplastic proliferation and differentiation of CNCCs were inhibited by cell-specific Nell-1 knockout, which is consistent with previous studies of primary cranial cells from END mice [55, 60]. Notably, compensation for Nell-1 deficiency through the administration of a recombinant gene can compensate for the deficiency in the osteogenic differentiation of CNCCs in Nell-1^Wnt1^KO mice. Therefore, Nell-1 is a key mediator of normal osteogenesis and is necessary for CNCCs to achieve their complete osteoplastic potential during craniomaxillofacial ossature growth [33]. More importantly, Nell-1 was strongly expressed in the dental follicles, and the fold change of expression is 4.16 in the DFs than in the PDL of human [7], which were detected by the cDNA microarray technique.

Nell‐1 has been reported to be a critical secretory protein in bone remodeling and osteoblastic cell differentiation and proliferation, as well as tooth development [61]. Numerous studies in animal models have shown the osteo-inductive activity of Nell-1 in accelerating bone regeneration [62, 63]. Animal models demonstrated the critical role of Nell‐1 in craniofacial bone growth. ENU-induced Nell-1-deficient newborn mice exhibited a defect similar to CCD in the calvarial bone [55], and the upregulation of Nell-1 expression in transgenic newborn mice (CMV-Nell-1) [57] resulted in CS symptoms. In addition to advancing the differentiation and mineralization of calvarial osteoblasts, Nell-1 can guide the regeneration and excessive growth of the skull [62]. Except for craniofacial bone [57, 64], deficient and compensatory Nell-1 mouse model indicated a major role of Nell-1 in the development of other bone tissue such as appendicular skeleton [65] and vertebra [66], as well as the growth of neural tissues [59]. Nell-1 also participates in the major process of both endochondral and intramembranous ossification [59]. The space and time specificity of Nell-1 expression in molar formation in mice [61] indicates that the development of ameloblasts and odontoblasts, the molar morphogenesis of crowns and roots, and the secretion and mineralization of the enamel and dentinal extracellular matrix require the participation of Nell-1. The wingless/integrated (Wnt)/*β*-catenin signaling pathway allows Nell-1 to bind to integrin *β*1 through its C-terminus, and the MAPK pathway allows Nell-1 to interact with Cntnap4 through its N-terminus to promote the formation of bone by osteoblasts [67], which was confirmed by recent data (see Fig. 2).

Nell-1 can promote the differentiation of different kinds of stem cells. In vivo and in vitro studies have shown that Nell-1 participates in the differentiation of dental pulp stem cells (DPSCs) into neural-like cells [68]. Human perivascular stem cells (hPSCs) expressing Nell-1 exhibited enhanced spinal fusion in osteoporotic rats [69] and accelerated chondrogenic differentiation [70]; the latter results may have occurred due to the increased responsiveness of hPSCs to BMPs + TGF-*β*s. In addition, Nell-1 is an osteoinductive differentiation factor that has been proven to promote mesenchymal stem cell (MSC) osteogenic differentiation [71]. Gingiva-derived stem cell spheroids showed increased osteogenic differentiation due to the role of Nell-1 [72]. Nell-1 was coexpressed with some neural markers, including neuron-specific enolase (NSE), substance P (SP) and Nestin, in the dental pulp of rats treated with Nell-1 as well as Nestin, *β*-III tubulin and glial fibrillary acidic protein (GFAP) in cultured hDPSCs treated with Nell-1 [68]. However, few studies of Nell-1 was conducted in hDFPCs though Nell-1 was detected to strongly express in DFs [7].

Thien et al. identified three OSE2 sites in the promoter of Nell-1 and suggested that Runx2 directly bound to the OSE2 sites and transactivated the human Nell-1 promoter [56], indicating that Nell-1 is a direct downstream target of Runx2 (see Fig. 2). Unlike other knockout model of downstream osteogenic gene of Runx2, animal knockout models [55] showed that loss of Nell-1 results in reduced thickness and density of calvarial bones in ENU-induced Nell-1–deficient (END) mice, similar to CCD-like calvarial phenotypes, in addition to rib cage and vertebral abnormalities [64]. Besides defective endochondral and intramembranous ossification in the experience, osteoblast markers including Runx2, osteocalcin and alkaline phosphatase was observed diminished [55]. These studies suggested that Nell-1 is an important downstream osteogenic gene of Runx2.

On the other hand, mice experiences [60] showed that Runx2-mediated osteoblastic gene expression and/or mineralization was severely reduced by Nell-1 siRNA oligos transfection into Runx2(+ / +) newborn mouse calvarial cells (NMCCs) or in N-ethyl-N-nitrosourea (ENU)-induced Nell-1(−/−) NMCCs. Nell-1 overexpression transgenic (CMV-Nell-1) mice partially the calvarial defects in the cleidocranial dysplasia (CCD)-like phenotype of Runx2(±) mice, whereas Nell-1 protein induced mineralization and bone formation in Runx2(±). Meanwhile, Nell-1 overexpression partially rescued osteoblastic gene expression but not mineralization in Runx2 null (Runx2(-/-)) NMCCs. Mechanically, Bokui et al. [73] found that Nell-1 can activate MAPK signaling pathways and thereby phosphorylate Runx2, resulting in the induction of human and rat osteoblast differentiation mechanistically. Similarly, Runx2 is overexpressed in nonsyndromic CS patients, which is considered to be a result of upregulated Nell-1 expression. These experiments show that Nell-1 can regulate Runx2 to some extent.

Osterix (Osx) is another well-studied downstream regulator of Runx2, a zinc finger-containing osteoblast-specific transcription factor that located restrictedly to the nucleus [74]. It has been proved that Osx acts downstream of Runx2/Cbfa1, since Runx2 is expressed in mesenchymal cells of Osx KO mice, while the expression of Osx is not observed in the lack of Runx2 [75]. Runx2 specifically binds to a DNA element at 99 nucleotides upstream of initiation codon of Osx gene in its promoter to take effect [76]. Two main way can lead to the induction of Osx gene expression, Runx2 dependent [77] or independent [78] pathway.

Chen et al. [79] suggested that with negative regulation of Nell-1, which is also tightly regulated by Runx2, Osterix is a direct transcriptional regulator contributing to a subtle balance of regulatory effects on Nell-1 transcription with Runx2, modulating Nell-1 expression levels as needed at different developmental time points. Overexpression of Osx result in significant decreased luciferase activity in Nell-1 promoter reporter systems and Nell-1 mRNA levels by specifically bind to Sp1 sites (Sp1/Osterix binding sites) within approximately 70 bp of the 5' flanking region of the human Nell-1 transcriptional start site. The study also showed that instead of competing with Runx2 binding to the OSE2 sites, Osterix downregulated Nell-1 by affecting binding of RNA polymerase II to the Nell-1 promoter. Recent study [80] also showed that by means of regulating Runx2/Osterix axis, activation of Nell-1 significantly improved osseointegration. Through Nell-1 could be inhibited by Osterix, overexpression of Nell-1 rescued the interference of gene and protein levels of Osterix, and Nell-1 had positive feedback regulation on the expressions of Runx2 and Osterix (see Fig. 2).

These studies suggested that Runx2/Nell-1 axis may act as one of the key regulatory pathways in DFPCs during the process of tooth eruption. However, many of the molecular experiments were conducted in other stem cells instead of hDFPCs. Lacking of enough studies on hDFPCs may make the grounds of argument not strong enough. Future studies conducted in the hDFPCs from CCD patients and healthy individuals are still needed for our better understanding the bone remodeling mechanism in hDFPCs and regulatory pathway of Runx2 and Nell-1.

### Posttranscriptional regulation

During gene expression, DNA is transcribed into messenger RNAs, which serve as blueprints for protein translation. Several classes of noncoding RNAs (ncRNAs) are necessary for these processes, which reveals the complexity of RNA-mediated regulation of gene expression [8]. MicroRNAs (miRNAs), which are encoded by endogenous genes, are a major class of small (18–25 nucleotides long) ncRNAs that bind to complementary sequences of target mRNAs, function in the posttranscriptional regulation of gene expression and suppress the expression of target mRNAs [9, 10]. After a series of processes including transcription, splicing and export, mature miRNAs directly interact with partially complementary target sites located in the 3′ untranslated region (3′ UTR) of target mRNAs through base pairing, leading to mRNA destabilization and translational repression [81, 82]. In addition, the process by which miRNAs regulate gene expression is complex, as miRNAs are able to target several mRNAs, and a number of miRNAs can target the same mRNA, suggesting a complex regulatory mechanism between miRNAs and mRNAs [83, 84]. Long noncoding RNAs (lncRNAs) are transcripts that are at least 200 nucleotides in length and have a poly-A tail; lncRNAs lack protein-coding potential [8, 85] and participate in several important life processes [86]. LncRNAs can localize to different places in the cell, and lncRNAs in the nucleus or cytoplasm function in different ways. Nuclear lncRNAs are primarily involved in gene regulation, such as epigenetic regulation of gene expression [87], regulation of nuclear architecture [88], and promoter-specific transcriptional regulation [89]. In contrast, lncRNAs localized to the cytoplasm mainly participate in posttranscriptional regulation gene of expression, including acting as miRNA sponges [90], controlling mRNA stability [91], and regulating miRNA biological signal transduction [92]. Circular RNAs (circRNAs) are covalently closed, endogenous biomolecules in eukaryotes with tissue-specific and cell-specific expression patterns, whose biogenesis is regulated by specific cis-acting elements and trans-acting factors. Some circRNAs are abundant and evolutionarily conserved, and many circRNAs exert important biological functions by acting as microRNA or protein inhibitors (‘sponges’), by regulating protein function or by being translated themselves [13].

The ceRNA hypothesis suggested a new mechanism underlying inter-RNA interactions [93]. As stated above, miRNAs suppress gene expression by binding to mRNAs, while competitive endogenous RNAs (ceRNAs), also termed miRNA sponges [12], regulate the expression of genes by competitively binding to miRNA response elements (MREs). As representative miRNA sponges in cells, lncRNAs or circRNAs contain multiple binding sites for one or several miRNAs and then titrate miRNAs away from their mRNA target genes, thereby regulating the posttranscriptional suppression of gene expression mediated by miRNAs and increasing the levels of target gene expression. This complex ceRNA network affects gene expression of osteoblast and osteoclast differentiation [8] and plays a key role in bone remodeling.

Many miRNAs and lncRNAs participant in the regulation of Runx2 for bone remodeling in a complex way, while few circRNAs was found to function in the process. MiR-221 negatively regulates Runx2 gene expression via its potential target sites in the 3'UTR of Runx2, thereby mediating osteoblast differentiation [94]. MiRNA-133a-5p targets the 3'UTR of Runx2, inhibiting its expression, osteoblast differentiation [95], and the expression of associated markers [e.g., collagen I, osteocalcin (OCN), and osteopontin (OPN)], extracellular matrix (ECM) mineralization, and alkaline phosphatase (ALP) activity [96]. MiR-133a targets the 3′UTR of Runx2 and inhibits osteoblast differentiation when overexpressed via the inhibition of alkaline phosphatase (ALP) [97]. MiR-223 and miR-19a can influence the RANKL-RANK pathway and the expression of MCP-1 by modulating the expression levels of TWIST and Runx2, thereby regulating the pathological process of osteolysis [98]. Overexpression of lncRNA GAS5 could promote the osteogenic differentiation of hMSCs by targeting microRNA-498 to upregulate Runx2 expression [99]. **L**ncRNA-DANCR was shown to recruit EZH2 to promote H3K27me3 and finally inhibit the transcription of the target gene Runx2 and suppress osteogenic differentiation [100]. LncRNA X-inactive specific transcript (XIXT) promotes the osteogenic differentiation of hBMSCs by targeting and sequestering miRNA-30a-5p and upregulating Runx2 expression [101]. LncRNA TSIX promotes osteoblast apoptosis by negatively regulating the expression of miR-30a-5p, knockdown of TSIX expression can inhibit Runx2 expression [102].

However, few scholars have studied the ceRNA network and subcellular regulation of Nell-1. MiRNA-370-3p and has-miR-485-5p were predicted to interact with circ0001543, circ0002405, and ENST00000570267 in ceRNA networks in Nell-1 induced osteogenic differentiation of human adipose-derived stem cells (hASCs) [103]. Besides, miR-370-3p was a key regulator in osteogenic differentiation of Nell-1 by targeting BMP2 and disturbing the expression of PTHLH [103]. Study [104] identified lncRNA and mRNA expression profiles during Nell-1 induced osteogenic differentiation of hASCs using high-throughput sequencing. Three core lncRNAs (ENST00000602964, ENST00000326734, and TCONS_00006792) were identified in CNC network may play an important role in Nell-1-induced osteogenesis of hASCs via the crosstalk between Hedgehog and Wnt pathways. RNA-sequencing identified two key circRNAs, namely circRFWD2 and circINO80, could regulate the expression of hsa-miR-6817-5p and have positive impact on the recombinant Nell-1-induced osteogenic differentiation of hASCs [105].

Since many studies investigated the ceRNA network of Runx2 and Nell-1 in subcellular level individually, non-study indicated the potential connection between Runx2 and Nell-1 in posttranscriptional level and precise regulation mechanism. Further research is still needed for digging deeper into the connection between Runx2 and Nell-1 in subcellular or posttranscriptional level of DFPCs, and other possible genes, molecules or regulatory factors involved in the Runx2/Nell-1 axis. Better understanding of the precise mechanism is also helpful for the development of potential clinical value of Runx2 and Nell-1 and better for targeted therapy of some diseases.

Though further researches are still needed, the possibility of clinical application of Runx2 and Nell-1 is gradually coming into our view based on the existing studies. As a novel, soluble growth factor with osteogenesis ability, Nell-1 can induce bone tissue denser, more calcified, and the positioning is more accurate compared with BMP-2 [106], indicating its biological advantages in the treatment of bone defects, and is a promising alternative to BMPs, attributable to its relative specificity to osteogenesis and less adverse effects. Nell-1 also improved implant osteointegration and showed the potential of Nell-1 gene therapy to shorten treatment time and broaden indications [80]. Besides being an important transcription factor in bone remodeling, Runx2 as a promising therapeutic target for cancers has become a research hotspot, since it plays a key role in the invasion and metastasis of cancers, and it is expected to become a new therapeutic target and contribute to the development of new drugs and the improvement of clinical efficacy [107]. With our deeper learning with Runx2 and Nell-1, their further clinical application is no longer out of reach.

## Conclusion

Cranial neural crest cells (CNCCs) can differentiate into dental follicles, who are necessary for tooth eruption and regulate both the formation and resorption of alveolar bone. Dental follicle progenitor cells (DFPCs) are stem cells found in dental follicles and differentiate into different kinds of cells that are necessary for tooth development and eruption. Being an important transcription factor, Runx2 is essential for osteoblast and osteoclast differentiation and bone remodeling, and it was proven to directly target downstream genes by binding with DNA element in its promotor to regulate its expression. Mutation of Runx2 causing cleidocranial dysplasia (CCD) negatively affects osteogenesis and the osteoclastic ability of dental follicles, resulting in tooth eruption difficulties. Among a variety of downstream targets of Runx2, Nel-like molecule type 1 (Nell-1) plays an important role in neural crest-derived tissues and strongly expressed in dental follicles. Nell-1 was originally identified in pathologically fused and fusing sutures of patients with unilateral coronal synostosis (UCS), and it plays indispensable roles in bone remodeling, including roles in osteoblast differentiation, bone formation and regeneration, craniofacial skeleton development, and the differentiation of many kinds of stem cells. Runx2 targets the Nell-1 gene and directly regulates its expression by binding to osteoblast-specific binding element 2 (OSE2) sites in the Nell-1 promoter. Osterix is a direct transcriptional regulator contributing to a subtle balance of regulatory effects on Nell-1 transcription with Runx2. These studies suggested that Runx2/Nell-1 axis may act as a key regulatory pathway in DFPCs during the process of tooth eruption. Studies on short and long regulatory noncoding RNAs have revealed the complexity of RNA-mediated regulation of gene expression at the posttranscriptional level. However, direct evidence of the role of Nell-1 in tooth eruption and the precise pathway has not been found, and these issues should be further investigated. This ceRNA network participate in the regulation of Runx2 and Nell-1 gene expression in a complex way. However, non-study indicated the potential posttranscriptional connection between Runx2 and Nell-1, further research is still needed. A more precise mechanism of action of Nell-1 and Runx2 not only helps us better understanding mode of regulatory network, but also allow us to explore their clinical significance.

## Data Availability

Not applicable.

## Abbreviations

DFPCs: Dental follicle progenitor cells
DFCs: Dental follicle cells
DFs: Dental follicles
CNCCs: Cranial neural crest cells
NELL-1/Nell-1: Nel-like molecule type 1
RUNX2/Runx2: Runt‐related transcription factor 2
ncRNAs: Noncoding RNAs
miRNAs: MicroRNAs
lncRNAs: Long noncoding RNAs
circRNAs: Circular RNAs
ceRNA: Competitive endogenous RNA
UCS: Unilateral coronal synostosis
CS: Craniosynostosis
CCD: Cleidocranial dysplasia
OSE: Osteoblast-specific binding element
OSE2: Osteoblast-specific binding element 2
BMP-2: Bone morphogenic protein 2
Wnt/*β*-catenin: Wingless/integrated/*β*-catenin
BMSCs: Bone mesenchymal stem cells
hPSCs: Human perivascular stem cells
MSC: Mesenchymal stem cell
DPSCs: Dental pulp stem cells
hDPSCs: Human dental pulp stem cells
CSF-1: Colony-stimulating factor 1
MMP: Matrix metalloproteinase
ALP: Alkaline phosphatase
Col1-*α*1: *α*1 Type I collagen
Bsp: Bone sialoprotein
Op: Osteopontin
Ocn: Osteocalcin
Osx: Osterix
DNMTs: DNA methyltransferases
MREs: MiRNA response elements
3′ UTR: 3′ Untranslated region
STAT1: Signal transducer and activator of transcription
